# Supplementary material for: P-TRAP: a Panicle Trait Phenotyping tool
Source: BMC Plant Biol. 2013 Aug 29;13:122. doi: 10.1186/1471-2229-13-122 (PMC3848748; doi:10.1186/1471-2229-13-122)
Supplement: Additional file 7 — P-TRAP source code. The Netbeans project that contains the source code of the application. [file 1471-2229-13-122-S7.zip › javadoc.zip!/javadoc/org-bioalg-skeletonprocessor/index-all.html]

Index (SkeletonProcessor)


JavaScript is disabled on your browser.


- Package
- Class
- Tree
- Deprecated
- Index
- Help

*org.bioalg.skeletonprocessor 1.0*

- Prev
- Next

- Frames
- No Frames

- All Classes

A B C D F G H N O R S T U V 


## A

addEndPoint(Point) - Method in class org.bioalg.skeletonprocessor.Skeleton


addIntersectionPoint(Point) - Method in class org.bioalg.skeletonprocessor.Skeleton


addSlapPoint(Point) - Method in class org.bioalg.skeletonprocessor.Skeleton


## B

buildGraph(Point, Point) - Method in class org.bioalg.skeletonprocessor.SkeletonProcessor
:   Recursively build a graph from the skeleton points


## C

calc01occurance(int[][]) - Method in class org.bioalg.skeletonprocessor.SkeletonExteractor


calcNonzero(int[]) - Method in class org.bioalg.skeletonprocessor.SkeletonExteractor
:   calculate the number of nonzero pixels.

calcNonzero(int[][]) - Method in class org.bioalg.skeletonprocessor.SkeletonExteractor


calcNonzero(int[][], int) - Method in class org.bioalg.skeletonprocessor.SkeletonExteractor


clean(int) - Method in class org.bioalg.skeletonprocessor.SkeletonExteractor


cleanimage(int, int) - Method in class org.bioalg.skeletonprocessor.SkeletonExteractor


clearSpikes(double) - Method in class org.bioalg.skeletonprocessor.Skeleton
:   Remove the spikes of a given length.


## D

deletepixels() - Method in class org.bioalg.skeletonprocessor.SkeletonExteractor


## F

firstsubiteration(int) - Method in class org.bioalg.skeletonprocessor.SkeletonExteractor


follow(Point, Skeleton, Skeleton) - Method in class org.bioalg.skeletonprocessor.SkeletonProcessor
:   Follow the skeleton recursively starting at a given point.

FromImage2GraphModel(File, int, boolean) - Static method in class org.bioalg.skeletonprocessor.GraphProducer
:   Convert the panicle in the image into a graph.


## G

getAllSkeletons(AImage, int) - Method in class org.bioalg.skeletonprocessor.SkeletonProcessor
:   Find all skeletons in a given skeleton image.

getAllSkeletons() - Method in class org.bioalg.skeletonprocessor.SkeletonProcessor
:   Return all skeletons from an image or a super skeleton.

getEndpoints() - Method in class org.bioalg.skeletonprocessor.Skeleton


getGraph() - Method in class org.bioalg.skeletonprocessor.SkeletonProcessor


getImage() - Method in class org.bioalg.skeletonprocessor.SkeletonExteractor


getIntersections() - Method in class org.bioalg.skeletonprocessor.Skeleton


getLength() - Method in class org.bioalg.skeletonprocessor.Skeleton


getNbyNsubmatrix(int, int, AImage, int) - Method in class org.bioalg.skeletonprocessor.SkeletonExteractor
:   return nXn matrix of the image

getNearst(Point, int) - Method in class org.bioalg.skeletonprocessor.Skeleton
:   Get the nearest point to the input point given a range.

getNeighbors(Point, int) - Method in class org.bioalg.skeletonprocessor.Skeleton
:   Get the list of neighbors of a point given a range threshold.

getNvector(int, int, AImage, int) - Method in class org.bioalg.skeletonprocessor.SkeletonExteractor


getPixelType(Point) - Method in class org.bioalg.skeletonprocessor.Skeleton


getPixelType(Point, int) - Method in class org.bioalg.skeletonprocessor.Skeleton


getPixelType(int[][]) - Method in class org.bioalg.skeletonprocessor.Skeleton


getSkeleton() - Method in class org.bioalg.skeletonprocessor.SkeletonProcessor


getSkeletonimg() - Method in class org.bioalg.skeletonprocessor.SkeletonProcessor


getSlaps() - Method in class org.bioalg.skeletonprocessor.Skeleton


getVisited() - Method in class org.bioalg.skeletonprocessor.SkeletonProcessor


GraphProducer - Class in org.bioalg.skeletonprocessor
:   Produce a graph from the the image representing it's skeleton

GraphProducer() - Constructor for class org.bioalg.skeletonprocessor.GraphProducer


## H

hotstair(int) - Method in class org.bioalg.skeletonprocessor.SkeletonExteractor


## N

NORTH - Static variable in class org.bioalg.skeletonprocessor.SkeletonExteractor


## O

org.bioalg.skeletonprocessor - package org.bioalg.skeletonprocessor


## R

removeEndPoint(Point) - Method in class org.bioalg.skeletonprocessor.Skeleton


removeIntersectionPoint(Point) - Method in class org.bioalg.skeletonprocessor.Skeleton


removeSlapPoint(Point) - Method in class org.bioalg.skeletonprocessor.Skeleton


removeSpikes(double) - Method in class org.bioalg.skeletonprocessor.SkeletonProcessor
:   Removes spikelets from the skeleton given a threshold

removesubmatrix(int, int, int) - Method in class org.bioalg.skeletonprocessor.SkeletonExteractor


removeTransitionVertices() - Method in class org.bioalg.skeletonprocessor.SkeletonProcessor
:   Removes any transition points

restVisited() - Method in class org.bioalg.skeletonprocessor.SkeletonProcessor


## S

setGraph(Graph) - Method in class org.bioalg.skeletonprocessor.SkeletonProcessor


setImage(AImage) - Method in class org.bioalg.skeletonprocessor.SkeletonExteractor


setSkeleton(AImage) - Method in class org.bioalg.skeletonprocessor.SkeletonProcessor
:   Set the skeleton from an image
    As different skeletons may exist in the image,
    this skeleton can be represented as main skeleton.

setSkeleton(Skeleton) - Method in class org.bioalg.skeletonprocessor.SkeletonProcessor


setSkeletonimg(AImage) - Method in class org.bioalg.skeletonprocessor.SkeletonProcessor


setVisited(ArrayList<Point>) - Method in class org.bioalg.skeletonprocessor.SkeletonProcessor


Skeleton - Class in org.bioalg.skeletonprocessor
:   Skeleton is represented as a sequence of points.

Skeleton() - Constructor for class org.bioalg.skeletonprocessor.Skeleton


Skeleton(AImage, int) - Constructor for class org.bioalg.skeletonprocessor.Skeleton
:   Classify the image pixels using a 3\*3 window to calculate the neighbors
    of a pixel if they are 1, it is an end point, >=2 an intersection , and a
    slap otherwise

Skeleton(ArrayList<Point>) - Constructor for class org.bioalg.skeletonprocessor.Skeleton
:   Create a skeleton form a set of points.

Skeleton.PIXEL\_TYPE - Enum in org.bioalg.skeletonprocessor


SkeletonExteractor - Class in org.bioalg.skeletonprocessor
:   Zhang-Suen algorithm in two different implementation.

SkeletonExteractor(AImage) - Constructor for class org.bioalg.skeletonprocessor.SkeletonExteractor


skeletonizeEXSZ() - Method in class org.bioalg.skeletonprocessor.SkeletonExteractor
:   Based on table implementation found here: http://rsbweb.nih.gov/ij/developer/source/ij/process/BinaryProcessor.java.html.

skeletonizeSZ() - Method in class org.bioalg.skeletonprocessor.SkeletonExteractor
:   The implementation of the SZ method based on the article specification.

SkeletonProcessor - Class in org.bioalg.skeletonprocessor
:   This is the main class.

SkeletonProcessor() - Constructor for class org.bioalg.skeletonprocessor.SkeletonProcessor


SOUTH - Static variable in class org.bioalg.skeletonprocessor.SkeletonExteractor


## T

tableBasedSZThining(int) - Method in class org.bioalg.skeletonprocessor.SkeletonExteractor
:   Based on table implementation found here: http://rsbweb.nih.gov/ij/developer/source/ij/process/BinaryProcessor.java.html.


## U

Unify(int) - Method in class org.bioalg.skeletonprocessor.SkeletonProcessor
:   Remove the corwoded nodes in the graph given a threshold


## V

valueOf(String) - Static method in enum org.bioalg.skeletonprocessor.Skeleton.PIXEL\_TYPE
:   Returns the enum constant of this type with the specified name.

values() - Static method in enum org.bioalg.skeletonprocessor.Skeleton.PIXEL\_TYPE
:   Returns an array containing the constants of this enum type, in
    the order they are declared.

A B C D F G H N O R S T U V

- Package
- Class
- Tree
- Deprecated
- Index
- Help

*org.bioalg.skeletonprocessor 1.0*

- Prev
- Next

- Frames
- No Frames

- All Classes
